# As Verified with the Aid of Biotinylated Spermine, the Brain Cannot Take up Polyamines from the Bloodstream Leaving It Solely Dependent on Local Biosynthesis

**DOI:** 10.3390/biom13071114

**Published:** 2023-07-13

**Authors:** Torsten Weiss, René Bernard, Gregor Laube, Julian Rieck, Misty J. Eaton, Serguei N. Skatchkov, Rüdiger W. Veh

**Affiliations:** 1 Institut für Integrative Neuroanatomie, Centrum 2, Charité—Universitätsmedizin Berlin, Corporate Member of Freie Universität Berlin and Humboldt-Universität zu Berlin, Charitéplatz 1, 10117 Berlin, Germany; torsten.weiss@charite.de (T.W.); gregor.laube@t-online.de (G.L.); 2 Excellenzcluster Neurocure, Charité—Universitätsmedizin Berlin, Corporate Member of Freie Universität Berlin and Humboldt-Universität zu Berlin, Charitéplatz 1, 10117 Berlin, Germany; rene.bernard@chdarite.de; 3 Institut für Zell- und Neurobiologie, Centrum 2, Charité—Universitätsmedizin Berlin, Corporate Member of Freie Universität Berlin and Humboldt-Universität zu Berlin, Charitéplatz 1, 10117 Berlin, Germany; julian.rieck@charite.de; 4 Department of Biochemistry, Universidad Central del Caribe, Bayamón, PR 00956, USA; mistyeaton1960@yahoo.com (M.J.E.); sergueis50@yahoo.com (S.N.S.); 5 Department of Physiology, Universidad Central del Caribe, Bayamón, PR 00956, USA

**Keywords:** polyamines, blood–brain barrier, local biosynthesis, metabolism, protoplasmic astrocytes, fibrous astrocytes

## Abstract

The importance of polyamines (PAs) for the central nervous system (CNS) is well known. Less clear, however, is where PAs in the brain are derived from. Principally, there are three possibilities: (i) intake by nutrition, release into the bloodstream, and subsequent uptake from CNS capillaries, (ii) production by parenchymatous organs, such as the liver, and again uptake from CNS capillaries, and (iii) uptake of precursors, such as arginine, from the blood and subsequent local biosynthesis of PAs within the CNS. The present investigation aimed to unequivocally answer the question of whether PAs, especially the higher ones like spermidine (SPD) and spermine (SPM), can or cannot be taken up into the brain from the bloodstream. For this purpose, a biotin-labelled analogue of spermine (B-X-SPM) was synthesized, characterized, and used to visualize its uptake into brain cells following application to acute brain slices, to the intraventricular space, or to the bloodstream. In acute brain slices there is strong uptake of B-X-SPM into protoplasmic and none in fibrous-type astrocytes. It is also taken up by neurons but to a lesser degree. Under in vivo conditions, astrocyte uptake of B-X-SPM from the brain interstitial fluid is also intense after intraventricular application. In contrast, following intracardial injection, there is no uptake from the bloodstream, indicating that the brain is completely dependent on the local synthesis of polyamines.

## 1. Introduction

Polyamines (PAs) may be derived from alimentary sources or local biosynthesis [1]. In the mammalian gut lumen, the major share of PAs stems from food intake, whereas a variable quantity may be produced by large intestine microbiota. PAs in the gut lumen are mainly absorbed by the duodenal and jejunal mucosa [2,3] and subsequently transferred [4] into the bloodstream (see Figure 1 in [1]). PAs in the gut lumen may reach almost millimolar concentrations after a meal and disappear rapidly and completely [3], whereby the luminal PA content returns to the fasting level in about 120 min [5]. Plasma levels of spermidine (SPD) and spermine (SPM) show only mild (up to 20 µM) increases after a meal [5]. Most likely PA uptake in peripheral tissues keeps the plasma concentrations low (see Table 2 in [1]).

Although most important biological functions of PAs in the CNS, such as regulation of receptors and ion channels, control of cell proliferation and differentiation, neuroprotection, anti-inflammation, or regulation of autophagia, are well known [6], it is not clear whether the brain receives its PAs from the bloodstream or whether it solely depends on local synthesis. Although a transport system for putrescine (PUT) has been demonstrated in brain slices [7], it was hardly detectable in vivo [8]. Experiments with radioactively labelled PAs indicated that the brain is unable to take up PAs from the bloodstream [9].

The present investigation aimed to unequivocally answer the question of whether PAs, especially the higher ones such as SPD and SPM, can or cannot be taken up from the bloodstream into the brain. For this purpose, we synthesized a biotin-labelled analogue of SPM (B-X-SPM) and used it as a tool to visualize this PA analogue in brain sections following its application to acute brain slices, to the bloodstream, or to the intraventricular space.

## 2. Materials and Methods

### 2.1. Materials

All chemicals and other materials were obtained from Sigma-Aldrich Chemie GmbH (82024 Taufkirchen, Germany) unless indicated otherwise.

#### 2.1.1. Synthesis of B-X-SPM

Biotinylated spacer-extended spermine (B-X-SPM) was synthesized following established methods to conjugate N-hydroxy-succinimide esters of haptens with amino group-containing molecules [10,11]. In short, 17.4 mg of spermine tetrahydrochloride and 27 μL of triethanolamine (to deprotonize SPM) were dissolved in 1 mL of dimethylformamide (DMFA; 50 mM SPM, solution A). Furthermore, 6.8 mg of 6-biotinamido-hexanoic acid N-hydroxysuccinimide ester (Molecular Probes) was dissolved in another 1 mL of DMFA (20 mM; solution B). For conjugation, 100 μL of solution A was combined with 150 μL of DMFA and supplemented with 250 μL of solution B. After 2 h at room temperature the reaction was stopped by the addition of 4.5 mL of pure ethanol, resulting in a 1 mM final concentration of biotinylated spermine.

#### 2.1.2. Characterization of B-X-SPM

**Figure 1:**
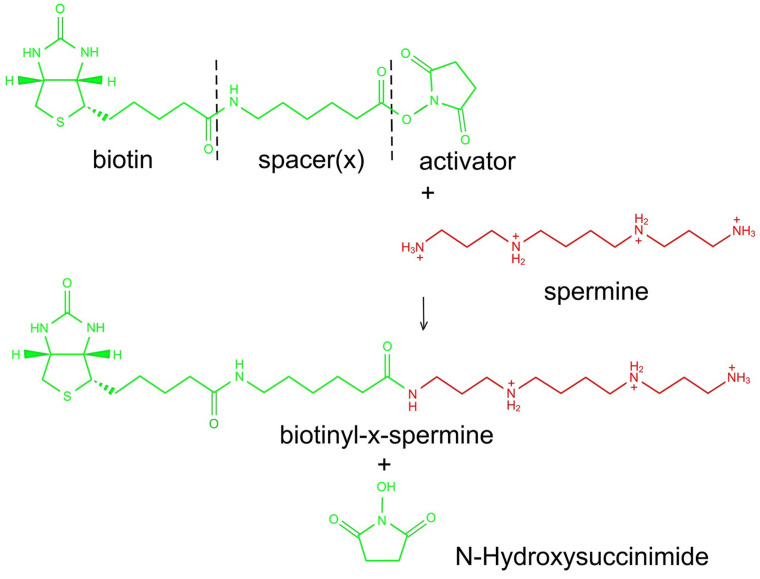
Synthesis of biotinylated and spacer-extended spermine (B-X-SPM).B-SPM was synthesized by directly coupling an extended and activated derivative of biotin (NHS-X-biotin; shown in green) to SPM (shown in red) as described in detail in the Methods section. Formation of B-SPM results in the release of N-hydroxy-succinimide.

Analysis and characterization of the reaction products were performed commercially (WITA GmbH, Berlin, Germany). Components of the reaction mixture were separated using reverse-phase high-pressure liquid chromatography, yielding two major peaks. The structure of the compound producing the first peak turned out to be N1-X-biotinyl-SPM (Figure 1), as confirmed by mass spectroscopy. The second peak most likely corresponds to N1,N12-doubly biotinylated SPM, as the presence of acylated secondary amino groups was ruled out by mass spectroscopy. This compound was not characterized in further detail.

#### 2.1.3. Solutions and Drugs

The saline solution (artificial CSF; aCSF) contained (in mM) 125 NaCl, 25 NaHCO_3_, 2.5 KCl, 1.25 NaH_2_PO_4_, 2 CaCl_2_, 2 MgCl_2_, and 25 D-glucose at pH 7.4 maintained by saturation with carbogen (95% O_2_/5% CO_2_). In the sucrose-aCSF used for brain preparation and during slicing, 50 mM sucrose was substituted for NaCl (final 75 mM). Spermine, biotinylated spermine (B-X-SPM), and betaine and carnitine, as organic cation transporter agonists, were dissolved as stock solutions and added to final concentrations in aCSF prior to application.

#### 2.1.4. Preparation of Acute Brain Slices

Brain slices were derived from adult male Wistar rats from an institutional breeding colony (Forschungseinrichtungen für Experimentelle Medizin, Charité-Universitätsmedizin Berlin, Germany). All experiments were approved by the Regional Berlin Animals Ethics Committee (T0127/02) and performed in strict accordance with the European Communities Council directive regarding care and use of animals for experimental procedures. All efforts were made to minimize the number of specimens and animal suffering.

Animals were deeply anesthetized with diethyl ether and decapitated. Brains were quickly dissected and placed into ice-cold sucrose-substituted artificial cerebrospinal fluid (aCSF). Coronal slices (300 µm) containing the habenular complex were cut using a vibrating microtome (VT 1000 S; Leica Instruments, Nussloch, Germany). Slices were placed in a holding vial, incubated in oxygenated aCSF (recording solution, see below) at 32–35 °C and continuously perfused with recording solution at a rate of 2.5–5.0 mL/min. Altogether 13 slices from 8 animals were necessary for the present investigation and a total number of 203 cryostat sections were analyzed.

#### 2.1.5. Superfusion of Brain Slices

For superfusion with selected drugs the aCSF solution was complemented with SPM (10 µM) or with B-X-SPM (5 to 20 µM). For competition experiments, in addition to B-X-SPM, the aCSF contained ethylenediamine, carnitine, betaine, or SPM, all at concentrations of 20 µM. Subsequent to superfusion, slices were briefly rinsed in aCSF and immediately fixed for 2 h in PGPic (see below). Thereafter they were cryoprotected (see below), mounted on a flat block of prefrozen Tissue Tek, shock-frozen in hexane at −70 °C, and stored at −80 °C until resectioning in a cryostat.

#### 2.1.6. In Vivo Injections

Animals were kept deeply anesthetized with isoflurane (Sigma-Aldrich, 792632). Rats were placed into a stereotaxic frame (David Kopf Instruments, Tujinga, CA, USA) and holes were drilled through the skull at selected positions for intracerebroventricular injections of B-X-SPM. Glass pipettes (tip diameter about 30 µm) were used for the injection of 8.0 or 25.0 µL of B-X-SPM in two separate animals yielding very similar results. Subsequent to injections, animals were fixed via transcardial perfusion with PGPic (4% paraformaldehyde, 0.05% glutaraldehyde, and 0.2% picric acid in 0.1 M phosphate buffer, pH 7.4 [12]). Brains, livers, and kidneys were removed, cryoprotected in 0.4 M sucrose for about 4 h and in 0.8 M sucrose overnight, cut into blocks, shock-frozen in hexane at −70 °C, and stored at −80 °C until use.

For intracardial injections of B-X-SPM, deeply anesthetized rats (3 animals) underwent thoracotomy under artificial respiration. After exposure, 300 µL of 2 mM B-X-SPM in PBS was injected into the heart using a conventional syringe. After a delay of 30 min the animals were fixed and treated as above.

#### 2.1.7. Immunocytochemistry

Freely floating cryostat sections (25 µm) were subjected to immunocytochemistry as described earlier [13]. In short, sections were rinsed in PBS (phosphate buffered saline; 150 mM sodium chloride in 10 mM phosphate buffer, pH 7.4), treated for 15 min with 1% sodium borohydride in PBS to remove residual aldehyde groups from the fixative, and again thoroughly washed in PBS. Sections were pretreated for 30 min in a blocking and permeabilizing solution (10% normal goat serum in 0.3% Triton X-100 and 0.05% phenylhydrazine in PBS) at room temperature (RT).

Anti-SPD/SPM antibodies [14] were applied for 36 h at appropriate dilutions in PBS containing 10% NGS, 0.3% Triton X-100, 0.1% sodium azide, and 0.01% thimerosal at 4° C. Sections were thoroughly rinsed in PBS, pretreated for 1 h with PBS-A (0.2% bovine serum albumin in PBS), and exposed for another 24 h at RT to the biotinylated goat anti-rabbit antibody (Vector Laboratories, Burlingame, CA, USA) diluted 1:2.000 in PBS-A containing 0.1% Triton X-100 and 0.1% sodium azide. After repeated washings in PBS and preincubation for one hour in PBS-A, the Elite avidin-biotin-peroxidase-complex (1:200 dilution in PBS-A; Vector Laboratories) was attached to biotinylated secondary antibodies by incubation for another 12 h at RT.

Next, sections were preincubated for 15 min in a solution of 0.5 mg/mL diaminobenzidine, 3 mg/mL ammonium nickel sulfate, and 10 mM imidazole in 50 mM Tris buffer, pH 7.6. Visualization of the antigen–antibody complexes was started by the addition of 0.0015% hydrogen peroxide and stopped after 15 min at RT by repeated washings with PBS. Sections were mounted onto gelatin-coated slides, air-dried for not longer than 30 min, dehydrated through a graded series of ethanol, transferred into xylene, and coverslipped with entellan.

#### 2.1.8. Selective Visualization of Biotinylated SPM in Tissues

The B-X-SPM molecule contains amino groups, which guarantee that it is retained in the tissue subsequent to aldehyde fixation. For visualization sections were pretreated as above. Subsequently they were directly exposed to the Elite avidin-biotin-peroxidase-complex (1:200 dilution in PBS-A; Vector Laboratories) and the B-X-SPM was visualized with diaminobenzidine as describe above [15].

## 3. Results

The present investigation aimed to understand where polyamines (PAs) in the mammalian central nervous system (CNS) are derived from. Principally, there are three possibilities: (i) intake by nutrition including the potential contribution from gut bacteria [1], release into the bloodstream from the gut, and subsequent uptake from CNS capillaries, (ii) products of general metabolism, release into the bloodstream from parenchymatous organs such as the liver, and again uptake from CNS capillaries, and (iii) uptake of precursors such as arginine [1] from brain capillaries and subsequent local biosynthesis of PAs within the CNS.

In the present investigation we focused on the most characteristic PA, spermine (SPM). Simulating uptake with natural SPM cannot produce unequivocal results, as the separate detection of endogenous and exogenously added PAs is hardly possible. Consequently, here we first prepared and characterized a biotinylated and extended analogue of SPM, called B-X-SPM. Subsequently, this SPM-analogue was used in brain slices and in vivo experiments to understand which parameters are important for SPM uptake in the mammalian brain.

### 3.1. Synthesis and Characterization of Biotinylated and Extended Spermine (B-X-SPM)

B-X-SPM (Figure 1) was synthesized by direct coupling an extended and activated derivative of biotin (NHS-X-biotin) to SPM as described in detail in the Methods section. Subsequently, the components of the reaction the mixture were separated using reverse-phase high-pressure liquid chromatography, yielding two major peaks. The structure of the compound producing the first peak turned out to be N1-X-biotinyl-SPM (Figure 1) as confirmed by mass spectroscopy. This is the active compound in our subsequent physiological experiments. The second peak most likely corresponds to N1,N12-doubly biotinylated SPM, as the presence of acylated secondary amino groups was ruled out by mass spectroscopy. This compound was not characterized in further detail.

### 3.2. In Vitro Uptake of Native or Biotinylated SPM in Brain Slices 

**Figure 2:**
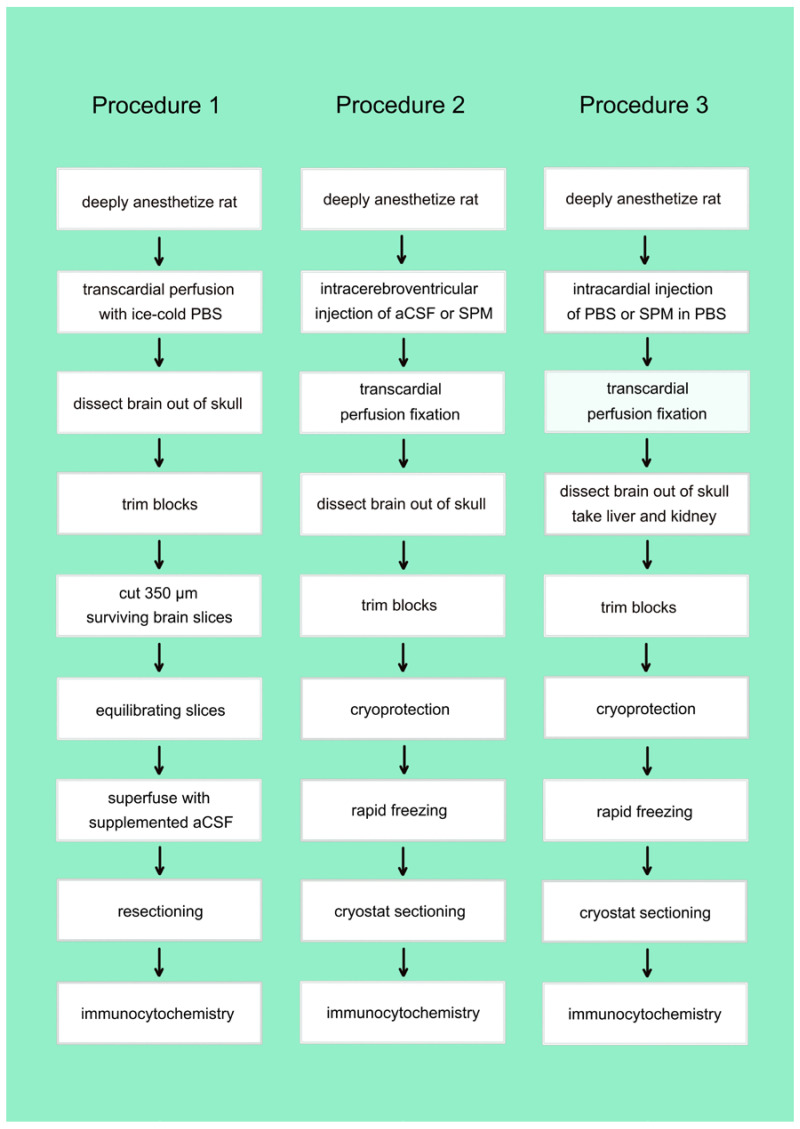
Procedures to visualize the potential uptake of SPM or B-X-SPM. Three distinct procedures were used to investigate, whether B-X-SPM is taken up by astrocytes of other brain cells from superfusing aCSF (procedure 1), from the brain extracellular space subsequent to intracerebroven-tricaular injection (procedure 2) or from the blood stream (procedure 3).

Brain slices provide a simple tool with which to simulate the uptake of native or biotinylated PAs from extracellular fluid. To verify this idea, in a first step we tested whether this system can be used to detect the uptake of native SPM (Figure 2; Procedure 1). SPM-containing artificial cerebrospinal fluid (aCSF) provides a constant source of PAs for all types of brain cells when it is used to superfuse acute brain slices.

### 3.3. Uptake of Native SPM in Acute Brain Slices 

**Figure 3:**
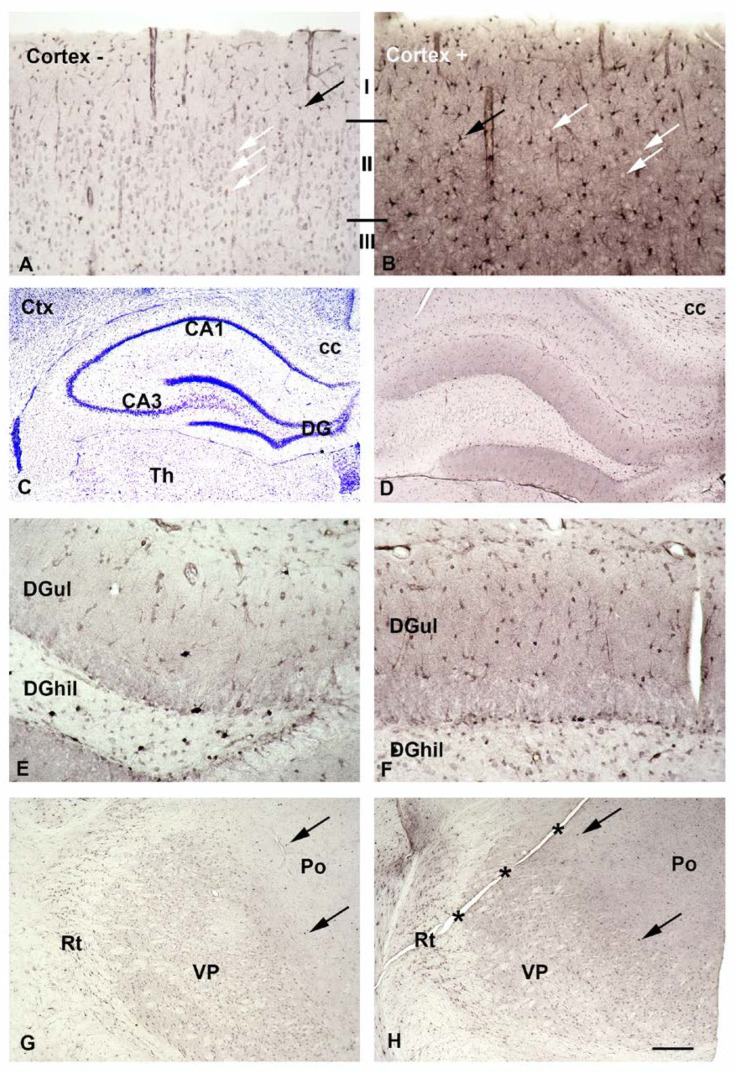
SPM-immunoreactivity in astrocytes and neurons after superfusion with aICF devoid of or complemented with SPM. Slices fixed immediately after sectioning (Ctx^−^) display astrocytes with prominent SPD/SPM-immunoreactivity in the cortex (**A**, black arrow). Neurons are positive also, but stained less intensely (**A**, white arrows). After equilibration (Ctx^+^), astrocytes retain strong immunoreactivity (**B**, black arrows), while neurons now are largely devoid of staining (**B**, white arrows), appearing as translucent circles in the positive neuropil. Roman numerals indicate cortical layers.A section stained with cresyl violet (**C**) is helpful to identify hippocampal regions (CA1, cornu ammonis 1; CA3.cornu ammonis 3; DG, dentate gyrus) and other areas (CTx, cortex; Th, thalamus; cc, Corpus callosum) in adjacent immunostained sections (**D** to **F**).Tiny dots detectable already in survey micrographs (**D**) and exemplarily labeled with arrows in (**G**,**H**) indicate that astrocytes retain SPM-immunoreactivity even after superfusion with SPM-free aCSF. They are most prominent (**D**, right upper corner) in the area of the Corpus callosum (labeled cc in **C** and **D**). After superfusion with SPM-containing aCSF staining is increased (**F**). When superfused with SPM-free aCSF, (**E**) the upper leaflet of the dentate gyrus (DGul) contains many weakly stained cells. After superfusion with SPM-containing aCSF most astrocytes display strong immunoreactivity here (**F**, DGul).Known GABAergic interneurons in the hilus of the dentate gyrus (DGhil) display only weak SPD/SPM-immunoreactivity (**E**,**F**), while inhibitory neurons in the reticular thalamic nucleus (Rt) are positive without (**G**) and even more after superfusion (**H**) with SPM. Neurons in the ventral posterior thalamic nucleus (VP) display also but weaker SPD/SPM-immunoreactivity (**G**,**H**), while the posterior thalamic nucleus (Po) is largely devoid of positive neurons (**G**,**H**), independent of whether the superfusion solution does (**H**) or does not (**G**) contain SPM. Astrocytes, however, are strongly positive (black arrows) even in the Po, while immunoreactivity of the neurpil is increased after superfusion with SPM (compare **G** to **H**). Asterisks identify a sectioning artifact. Bar in (**H**) indicates 540 µm in (**C**), 300 µm in (**D**,**G**,**H**), and 70 µm in (**A**,**B**,**E**,**F**).

In a first set of experiments (Figure 3; the Nissl stain, 3C, is included to help people who are not very familiar with the substructures of the hippocampus to orientate themselves in the other sections), we asked whether intracellular SPM might be lost during pretreatment of acute slices.

When slices are fixed immediately after sectioning astrocytes display prominent SPD/SPM-immunoreactivity. Neurons are also positive, but stain less intensely (Figure 3A). After 90 min of equilibration, astrocytes retain strong immunoreactivity, whereas neurons now appear as translucent spots (Figure 3B), suggesting that the PAs have been lost.

Tiny dots already detectable in survey micrographs (Figure 3D) indicate that astrocytes retain SPM-immunoreactivity even after superfusion with SPM-free aCSF. They are most prominent (Figure 3D, right upper corner) in the area of the corpus callosum (labeled cc in Figure 3C). Staining becomes more intense when brain slices are superfused with SPM-containing aCSF, most prominently in the hippocampal dentate gyrus (DG, compare Figure 3E,F). This higher magnification also verifies that the tiny dots in Figure 3D mostly represent astrocytes.

Surprisingly, separate types of neuron, or neurons in distinct areas, may accumulate PAs to different levels. Thus, known GABAergic interneurons in the hilus of the dentate gyrus display weak SPD/SPM-immunoreactivity (Figure 3E,F). In contrast, inhibitory neurons in the reticular thalamic nucleus (Rt) are positive without, and even more when superfused with, SPM (Figure 3G,H). Neurons in the ventral posterior thalamic nucleus (VP) display weaker SPD/SPM-immunoreactivity (Figure 3G,H), whereas the posterior thalamic nucleus (Po) is largely devoid of positive neurons (Figure 3G,H), independently of whether the superfusion solution does or does not contain SPM. Astrocytes, however, are strongly positive even in the Po, whereas immunoreactivity of the neuropil is increased after superfusion with SPM (compare Figure 3G–H).

### 3.4. Uptake of Biotinylated SPM (B-X-SPM) in Acute Brain Slices 

The use of biotinylated SPM (B-X-SPM; see Figure 1) enabled us to visualize SPM freshly taken up in the presence of SPM which was already in the cell. For this purpose, acute slices were superfused with B-X-SPM, subsequently resectioned, and used for immunocytochemistry (see Figure 2). Total SPM was demonstrated with our anti-SPM antibody [14], whereas staining directly with the ABC complex selectively visualizes the freshly internalized biotinylated SPM.

**Figure 4:**
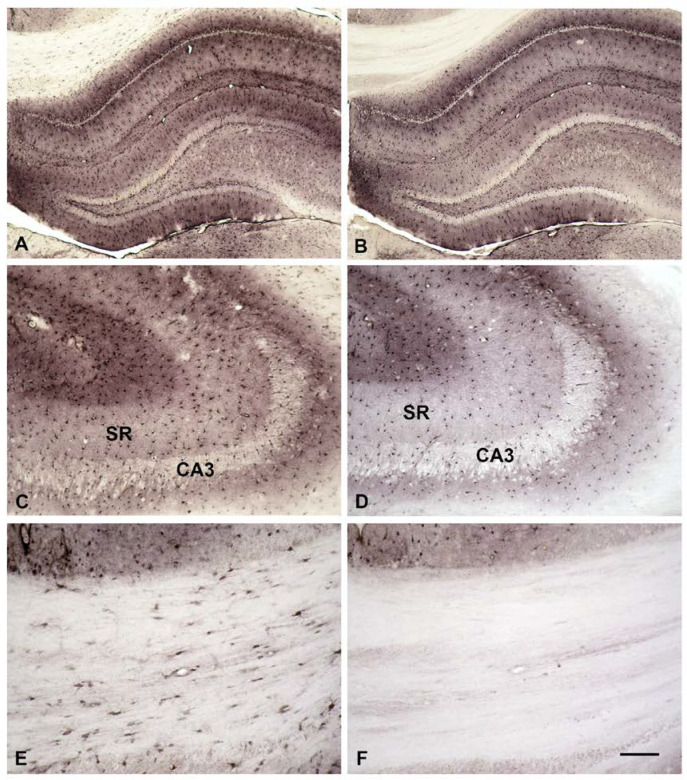
SPM, which has been freshly taken up by astrocytes, is visualized in the presence of SPM which was already in the cell. Biotinylated SPM freshly taken up by the acute slice is visualized by direct application of the ABC complex even in the presence of SPM, which had been in the cell before. Total SPM was demonstrated with our anti-SPM antibody. At the first glance both stainings appear rather similar (**A**,**B**). Closer inspection however, might lead to the impression that suggests that more SPM-positive cell bodies are detected by the anti-SPM antibodies (**A**,**C**) as compared to the ABC complex alone (**B**,**D**). This, however, is not the case. Instead, the cells contain more total SPM as evident by the prominently stained astrocyte processes with the anti-SPM antibody (**C**). These are less easily detectable, when only the newly incorporated B-X-SPM is visualized (**D**). Not all astrocytes are able to take up B-X-SPM. Fibrous-type astrocytes in the Corpus callosum do present SPM-like immunoreactivity (**E**), but remain negative for newly uptaken B-X-SPM (**F**). Bar in (**F**) indicates 300 µm in (**A**,**B**), 230 µm in (**C**,**D**), and 70 µm in (**E**,**F**).

At first glance both images appear rather similar (compare Figure 4A,B). At higher magnification, however, one might get the impression that, especially in the stratum radiatum (SR), more SPM-positive cell bodies are detected by the anti-SPM antibodies (Figure 4C) than by the ABC complex alone (Figure 4D). This, however, is not the case and the total number of astrocytes in this region shows no obvious difference between the two images. Instead, the cells contain more total SPM as evident by their prominently stained processes (Figure 4C). These processes are less easily detected when only the newly incorporated B-X-SPM is visualized (Figure 4D).

In fact, not all astrocytes are able to take up B-X-SPM. Thus, the fibrous-type astrocytes in the corpus callosum do present SPM-like immunoreactivity (Figure 4E), but they remain negative for newly uptaken B-X-SPM (Figure 4F). Apparently, fibrous-type and protoplasmic-type astrocytes are quite distinct in their ability to take up PAs from the surrounding intercellular fluid.

### 3.5. In Vivo Uptake of Native or Biotinylated SPM into the Brain from Intraventricular CSF or from the Bloodstream

Our data so far demonstrate that B-X-SPM is a good surrogate with which to visualize the uptake of the PA SPM from the surrounding brain intercellular space into the brain.

### 3.6. Incorporation of B-X-SPM into Astrocytes Is Based on a PA-Specific Uptake System

Uptake of B-X-SPM as a surrogate for SPM, as visualized above, might be due to any more or less specific cation transporter. To support the idea that this uptake is based on a PA-specific system, non-PA potential competitors were co-applied with B-X-SPM. When betain, carnitine, or ethylenediamine were used as competitors, B-X-SPM uptake was as strong as if no competitor was present. In contrast, when the slice was superfused with additional 20 mM SPM, uptake of B-X-SPM could not be detected any longer (Supplementary Figure S1).

### 3.7. In Vivo Uptake of Biotinylated SPM into the Brain from Intraventricular CSF 

**Figure 5:**
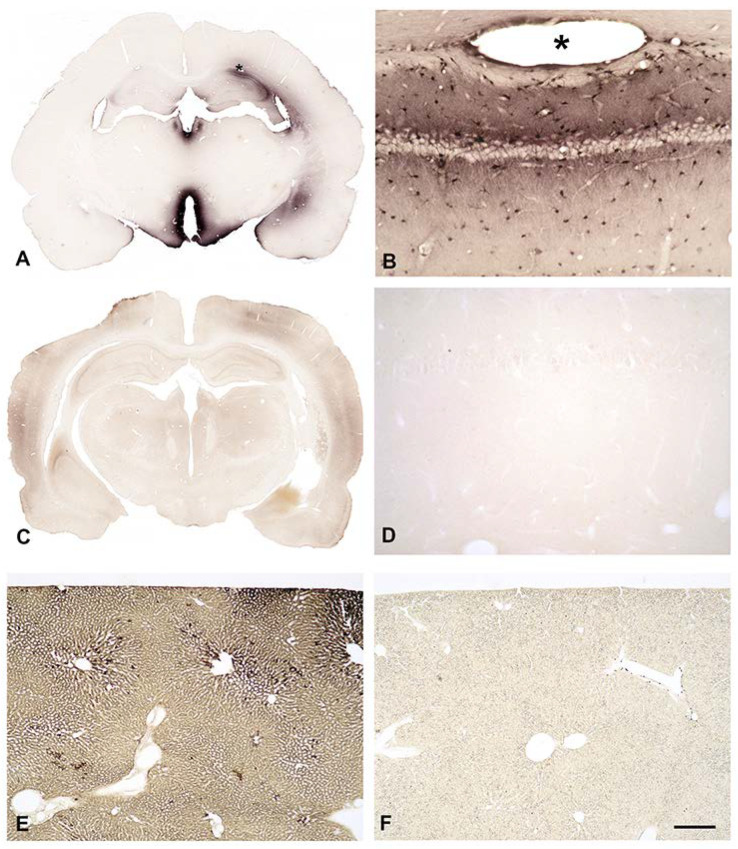
In vivo uptake of biotinylated SPM from intraventricular CSF or from the bloodstream. The ABC complex is used to detect B-X-SPM injected into the right lateral cerebral ventricle of deeply anesthetized rats (**A**,**B**; asterisks indicate the positions of the suprahippocampal recessus). Ventricles, especially the third one, are surrounded by strong staining for B-X-SPM (**A**). Higher magnification of the CA1 hippocampal area displays positive neuropil and strongly stained astrocytes (**B**). After intracardial injection of B-X-SPM, no uptake of B-X-SPM into the CNS is observed (**C**). Even at high magnification no immunoreactivity can be detected (**D**). However, B-X-SPM is strongly taken up by the liver (**E**), indicating that it can be taken up from the bloodstream. The liver of a control animal that did not receive B-X-SPM, is largely negative (**F**). The bar indicates 1700 µm in (**A**,**C**,**F**), 260 µm in (**E**,**F**), and 100 µm in (**B**,**D**).

To learn whether astrocytes do collect Pas such as SPM from the intercellular fluid, we injected B-X-SPM into the right lateral cerebral ventricle of deeply anesthetized rats. Apparently, the material moved/diffused along the left lateral ventricle, finally reaching the third ventricle (Figure 5A). In addition to the left hippocampal subfields there is some uptake in the area of the amygdala, in the habenula, and in the paraventricular thalamic nucleus. Higher magnification of the CA1 hippocampal area displays positive neuropil and strongly stained astrocytes (Figure 5B). The pronounced uptake in the hypothalamic periventricular zone is most likely to be due to local tanicytes.

### 3.8. In Vivo Uptake of Biotinylated SPM into the Brain from the Bloodstream 

To learn whether astrocytes do collect SPM from the bloodstream, we injected a single dose of B-X-SPM intracardially in deeply anesthetized rats. After 30 min, animals were euthanized by transcardial fixation and brain, liver and kidneys were subjected to immunocytochemistry.

Surprisingly, no uptake of B-X-SPM into the CNS could be observed (Figure 5C). Minor background staining (Figure 5C) results when sections adhere for some time to the bottom of the well during incubation. Higher magnifications of the darker Ctx region from Figure 5C confirm background staining.

At higher magnification absolutely no immunoreactivity can be detected (Figure 5D). However, B-X-SPM is easily taken up by liver (Figure 5E) and kidney, indicating that, in peripheral organs, polyamines can indeed be taken up from the bloodstream.

## 4. Discussion

Our newly synthesized biotinylated and spacer-extended spermine (B-X-SPM) had already been used in a previous investigation [15]. Here it is characterized in more detail and examined as invaluable tool to follow the uptake of polyamines (PAs), especially spermine (SPM), by distinct cell type and organ using in vitro and in vivo conditions.

### 4.1. Technical Considerations

For coupling biotin to SPM we used an N-hydroxysuccinimide activated and spacer-extended biotin derivative and native SPM (Figure 1). As SPM contains two primary and two secondary amino groups it was unclear which of these two groups would be attacked by the activated SPM derivative. Reverse-phase chromatography (see methods section) of the reaction products resulted in two major peaks. The first one represented the desired compound (Figure 1), as characterized by mass spectroscopy. The second one most likely contained a doubly coupled SPM and was not investigated further for economic reasons. Using either the material in the first peak or the complete mixture after reaction in pilot experiments for our biological studies revealed no major differences. Consequently, the complete mixture was used in all subsequent attempts.

B-X-SPM certainly only represents a surrogate for native SPM or other polyamines. Our competition studies, however, indicate that its uptake is based on the same system as that of natural SPM and, most likely, that of other Pas, such as SPD, as well.

### 4.2. Native and Biotinylated SPM Are Taken up by Astrocytes and Neurons in Brain Slices 

Astrocytic cells are not able to synthesize PAs on their own [16] but accumulate them much more effectively than neurons [1,14,17,18,19]. In contrast, neurons do synthesize PAs, most likely from arginine taken up from the bloodstream [1]. They express ornithine decarboxylase [20,21] and Spd/SPM synthase [16] and also display SPD/SPM immunoreactivity [22].

Interestingly, neuronal SPD/SPM immunoreactivity largely differs between neuronal areas and cell types. Here we find that neuronal staining is weak in the cerebral cortex and in the hippocampus (see Figure 3). Even interneurons in dentate hilus largely are devoid of immunostaining, whereas inhibitory neurons in the reticular thalamic nucleus are clearly SPD/SPM-positive, and this positivity is even increased after preincubation with native SPM. In contrast, in the same section, neurons in the ventral posterior thalamic nucleus display weak staining only, and in the posterior thalamic nucleus no staining is evident at all (see Figure 3G,H). At first glance such differences are surprising, but have also been described earlier [22].

### 4.3. Uptake of Biotinylated SPM (B-X-SPM) by Brain Slices Is Different in Protoplasmic and Fibrous Astrocytes

Uptake of B-X-SPM into brain slices is due to a specific cation transporter system [15], which is also supported here. Competing uptake by native SPM completely abolished the appearance of B-X-SPM in brain slices, whereas betain, carnitine, and ethylenediamine produced no effects (Supplemental Figure S1).

Most interestingly, not all types of astrocyte can easily take up B-X-SPM. Astrocytes in the corpus callosum, generally known to belong to the fibrous type, are apparently not able to take up B-X-SPM easily (see Figure 4E,F). The importance of this old division of astrocytes into protoplasmic and fibrous types has recently been supported by novel data [23]. Experiments with transgenic mice indicate that the Hedgehog pathway, well known for its role in the developing CNS, is active in astrocytes in the mature mouse forebrain. Not all astrocytes, however, respond to Sonic hedgehog (Shh), which in responsive cells activates Gli1 to achieve its effects. Gli1-expressing astrocytes display a morphology corresponding to the protoplasmic type of astrocyte, whereas fibrous-type astrocytes are not affected by Shh [23]. The importance of this fact is evident, taking into account that neurons are the source of Shh. Apparently, only protoplasmic-type astrocytes can respond to this neuronal communication whereas fibrous-type cannot [23]. This is not surprising when one considers that fibrous astrocytes mostly reside in the white matter, where neurons are extremely rare. The mouse data above, in combination with ours, give further support to the idea that protoplasmic and fibrous-type astrocytes belong to different subfamilies with potentially distinct biological functions.

### 4.4. Native or Biotinylated SPM Are Not Taken up into the Brain from the Bloodstream

Subsequent to its intraventricular injection, B-X-SPM is easily detected in the surrounding brain areas (Figure 5A,B), indicating unhindered uptake of PAs from the CSF. In contrast, following intracardial injection, no B-X-SPM could be detected in the parenchyma of the brain. Consequently, our data strongly favor the idea that, in line with earlier indirect evidence [7,8,9], the brain is not able to take up higher PAs from the bloodstream.

## 5. Conclusions

A biotinylated SPM analogue (B-X-SPM) was found to be an invaluable tool for investigating the uptake of PAs, especially SPM, by distinct cell type and organ using in vitro and in vivo conditions. In acute slices, uptake of B-X-SPM is strong in protoplasmic and absent in fibrous-type astrocytes. It is also taken up by neurons, but to a lesser degree. Under in vivo conditions, astrocyte uptake of biotinylated SPM from the brain interstitial fluid is also strong after intraventricular application. In contrast, following intracardial injection, there is no uptake from the bloodstream. Earlier experiments with radioactively labeled PAs [9], together with the present data, strongly suggest that the brain is largely dependent on the local synthesis of polyamines.

## Data Availability

Data supporting reported results are available from the senior author on request.

## Supplementary Materials

The following supporting information can be downloaded at: https://www.mdpi.com/article/10.3390/biom13071114/s1, Figure S1. Competition experiments indicate that uptake of B-X-SPM is based on a PA-specific system.
