# Supplementary material for: As Verified with the Aid of Biotinylated Spermine, the Brain Cannot Take up Polyamines from the Bloodstream Leaving It Solely Dependent on Local Biosynthesis
Source: Biomolecules. 2023 Jul 13;13(7):1114. doi: 10.3390/biom13071114 (PMC10377363; doi:10.3390/biom13071114)
Supplement: Supplementary file 1 [file biomolecules-13-01114-s001.zip › biomolecules-2435133-supplementary.pdf]

biomolecules

MDPI

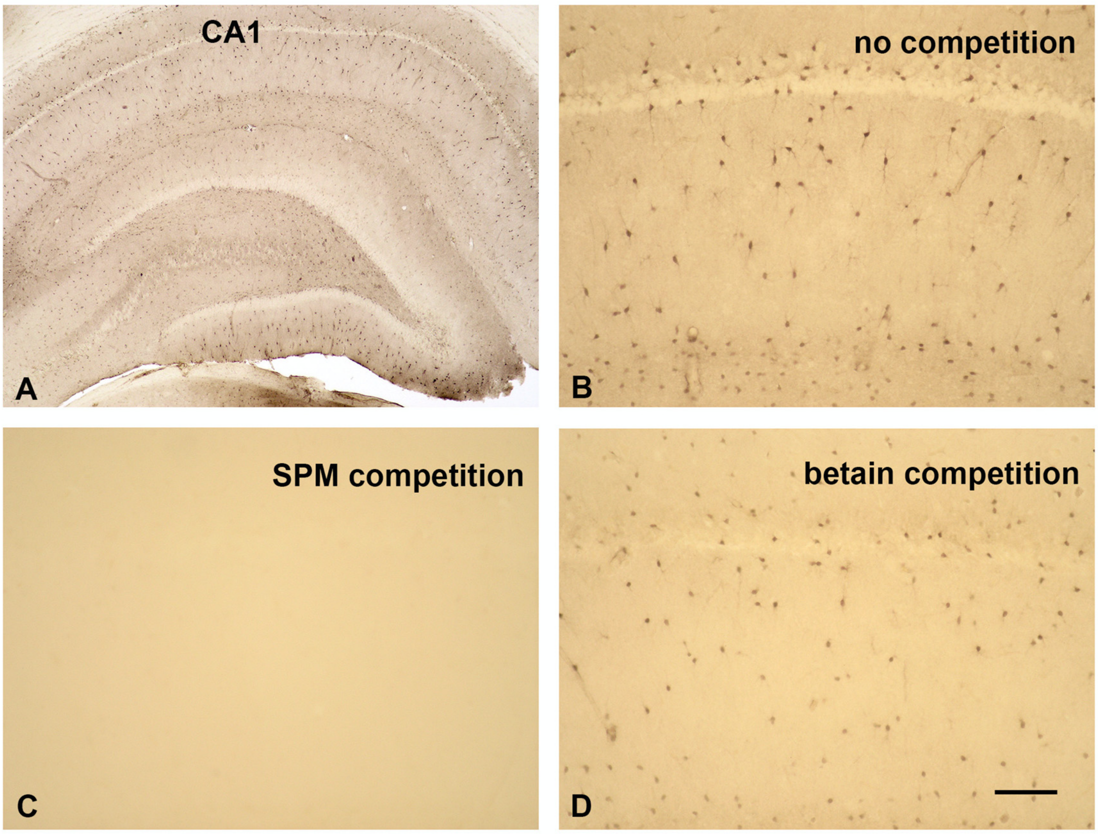

Supplementary Figure

Figure S1. Competition experiments indicate that uptake of B-X-SPM is based on a PA-specific system. When the uptake of B-X-SPM is due to a PA-specific system, added native PAs, but not other cations, must act as competitors, reducing the uptake of B-X-SPM. This fact is documented here. (A) When acute rat brain slices are superfused with artificial CSF containing B-X-SPM, it is eagerly taken up by astrocytes. They appear as tiny dots in survey micrographs of the hippocampus. (B) Higher magnification of the hippocampal CA1 area verifies that the small dots indeed are astrocytes. (C) When the superfusion fluid contains an excess of SPM in addition to B-X-SPM, uptake is abolished completely. (D) The presence of excess betain addition to B-X-SPM does not result in any visible competition. Bar indicates 300 µm in (A) and 70 µm in (B), (C), and (D).
